# Influences of Increasing Pedicle Screw Diameter on Widening Vertebral Pedicle Size during Surgery in Spinal Deformities in Children and Adolescents without Higher Risk of Pedicle and Vertebral Breaches

**DOI:** 10.3390/jcm12165368

**Published:** 2023-08-18

**Authors:** Pawel Grabala, Ilkka J. Helenius, Michal Grabala, Suken A. Shah

**Affiliations:** 1 Department of Pediatric Orthopedic Surgery and Traumatology, University Children’s Hospital, Waszyngtona 17, 15-274 Bialystok, Poland; 2 Department of Orthopedics and Traumatology, Helsinki University Hospital, 00260 Helsinki, Finland; ilkka.helenius@helsinki.fi; 3 2nd Clinical Department of General and Gastroenterogical Surgery, Medical University of Bialystok, ul. M. Skłodowskiej-Curie 24a, 15-276 Bialystok, Poland; michal@grabala.pl; 4 Department of Orthopaedic Surgery, Nemours Children’s Health, Delaware Valley, 1600 Rockland Road, Wilmington, DE 19803, USA; suken.shah@nemours.org

**Keywords:** pedicle screw, increasing screw diameter, scoliosis, spinal deformity, screw placement, pedicle expanding

## Abstract

Background: A very common technique for treating spinal deformities in children and adolescents is the use of segmental screws. In order to obtain proper stability and the best possible correction, the screws must first be precisely inserted. Additional factors influencing the quality and success of the operation are the size and quality of the bone, the skills of the surgeon, and biomechanical factors, i.e., the width and length of the screws used during surgery. Our study was focused on evaluating the effect of increasing the diameter of the instrumented pedicles by pedicle screws and assessing the safety of expanding these pedicles with screws of various sizes in children with spinal deformities during the growth period, using preoperative magnetic resonance imaging and postoperative computed tomography (CT) to assess and compare preoperative size measurements from MRI to postoperative CT measurements. Methods: We obtained data for evaluation from the available medical records and treatment histories of patients aged 2 to 18 who underwent surgical treatment of spinal deformities in the years 2016–2023. In 230 patients (28 male and 202 female), 7954 vertebral bodies were scanned by preoperative MRI, and 5080 pedicle screws were inserted during surgery, which were then assessed by postoperative CT scan. For the most accurate assessment, patients were classified into three age groups: 2–5 years (Group 1), 6–10 years (Group 2), and 11–18 years (Group 3). In addition, we studied implant subgroups: vertebral bodies with inserted pedicles of screw sizes 5.0 mm and 5.5 mm (Group S), and pedicles of screw sizes 6.0 mm, 6.5 mm, and 7.0 mm (Group L). Results: The morphology of pedicles (Lenke classification) analyzed before surgery using MRI was 55.2% type A, 33.8% type B, 4.7% type C, and 6.3% type D. The postoperative lateral and medial breaches were noted, and these did not cause any complications requiring revision surgery. The mean pedicle diameter before surgery for T1–L5 vertebral pedicles was between 3.79 (1.44) mm and 5.68 (1.64) mm. The mean expanding diameter of pedicles after surgery for T1–L5 vertebral pedicles ranged from 1.90 (0.39) mm to 2.92 (0.28) mm, which corresponds to the extension of the pedicle diameter in the mean range of 47% (4.1)–71% (3.0). We noted that the mean vertebral pedicle expansion was 49% in Group 1, 52% in Group 2, and 62% in Group 3 (N.S.), and the mean expansion for 7.0 mm screw pedicles was 78%. Conclusions: Our study confirms that there is a wide range of expansion of the vertebral pedicle during screw insertion (up to 78%) with a low risk of lateral or medial breaches and without an increased risk of complications. The larger the diameter of the screw inserted into the pedicle, the more the pedicle expands. Pedicle measurements by preoperative MRI may be helpful for sufficient reliability in preoperative planning.

## 1. Introduction

Treatment of spinal deformities in children and adolescents is currently impossible without the use of pedicle screws [1,2,3]. The literature describes the morphology of pedicles and the frequency of occurrence of their types in the population, which has a significant impact on the safety of screw insertion and subsequent correction [4,5,6,7,8,9,10,11,12,13,14,15]. Many scientific studies on the biomechanics of screws have proven that, in addition to the selection of appropriate screw parameters, increasing the diameter of the screw so that it optimally fills the pedicle will result in the best stability and resistance of the screw to the effect of pulling out and breaking [16,17,18,19,20,21,22,23,24]. Compared with adult patients, children’s spines may be more flexible, and thus the pedicles can widen to accommodate larger-diameter screws [21,25,26]. So far, several studies have been conducted on human cadavers, proving the possibility of widening the vertebral pedicles with the use of larger pedicle screws [23,27]. Similar studies have also been conducted on non-human animals, and authors have reported a vertebral pedicle expansion phenomenon in a population of pigs with immature pedicles [28,29]. These studies show that a large-diameter screw allows for a stronger fixation without compromising the spinal canal. However, there are no clinical trials evaluating the feasibility and safety of large screws for deformities in human children and adolescents. In addition, the use of a larger screw size allows for better correction of a three-dimensional spinal deformity and better reconstruction of the sagittal and coronal balance of the spine; however, there are no other reports in the literature describing radiological outcomes of inserting larger pedicle screws in children during growth or the influence of larger-sized screws on pedicle widening.

In our study, we analyzed the use of larger transpedicular screws in the surgical treatment of spinal deformities in children and adolescents during growth. We recorded the effect of increasing the diameter of the screws on the vertebral pedicles. At the same time, we tried to assess the safety of pedicle expansion with these screws as well as identify possible complications. All measurements were made from preoperative magnetic resonance imaging (MRI) and postoperative computed tomography (CT) data in order to compare the results of pedicle screw insertion in children and adolescents. We hypothesized that pediatric pedicles can safely be expanded using larger pedicle screws to maximize the screw purchase in the instrumented vertebrae.

## 2. Materials and Methods

### 2.1. Setting and Patients

We obtained approval from the institutional review board for this retrospective analysis. We used the available medical documentation and the history of surgical treatment of spinal deformities in patients aged 2 to 18 who were surgically treated with various posterior surgical techniques with the use of transpedicular screws in the years 2016–2023 in a pediatric spine center, with techniques described in the literature [1,2,3,30]. Patients with full medical documentation, a complete medical history, radiological images, a preoperative MRI of the entire spine, and a postoperative CT scan were selected for the study. In total, 230 patients (28 males and 202 females), including 7954 vertebral pedicles, were scanned by preoperative MRI, and there were 5080 pedicle screws inserted during surgery, which were subsequently evaluated with postoperative CT scans. Two independent spinal surgeons reviewed the patient histories and measurements. All patients whom we deem to qualify for spinal surgery and those operated on due to spinal deformities must undergo an MRI examination of the entire spine before surgery to accurately diagnose potential concomitant defects. We do not routinely perform a preoperative CT scan due to high radiation doses. Our most common indications for postoperative CT are usually severe spinal deformity, congenital scoliosis, missing or dysplastic pedicles, postoperative back pain or a new neurological deficit, suspected screw perforation, or a misplaced screw on postoperative radiographs. All CT scans were performed immediately after surgical correction or after the appearance of a critical symptom following index surgery. The inter- and intra-observer variability was calculated and evaluated using the Kappa (k) method. The analyzed patients were classified into three groups: patients aged 2–5 years (Group 1), 6–10 years (Group 2), and 11–18 years (Group 3). In addition, we studied implant subgroups: vertebral bodies with inserted pedicle screw sizes of 5.0 mm and 5.5 mm (Group S), and pedicle screw sizes of 6.0 mm, 6.5 mm, and 7.0 mm (Group L). The etiologies for all patients were idiopathic, congenital, syndromic, and neuromuscular. A subgroup analysis of the increasing pedicle diameter for vertebral pedicle widening and vertebral breaches in the groups was performed. Preoperative details, operation and instrumentation details, and other demographic data, including complications, were noted from the charts (Table 1).

### 2.2. Outcome Parameters

All patients selected for this study were treated using pedicle screws during surgery with spinal fusion (PSF) or surgery without fusion with growing rod techniques or growth guidance systems. Each operation was performed by a team of two experienced spine surgeons or one spine surgeon and a neurosurgeon with appropriate experience in spinal deformity surgery in children and adolescents. All patients underwent intraoperative spinal cord monitoring, including somatosensory evoked potentials (SSEP) and transcranial motor evoked potentials (MEP) [31,32,33]. We analyzed the number and size of the screws used, and we assessed the size of the pedicles before and after the surgery, after screw implantation. We recorded the number of segment screws used, the number of implanted levels, and potential complications requiring secondary treatment or revision procedures.

### 2.3. Radiographic Parameters

**Figure 1:**
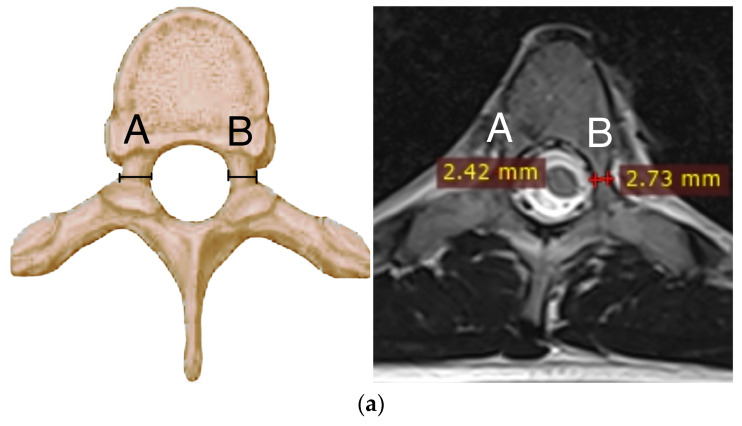

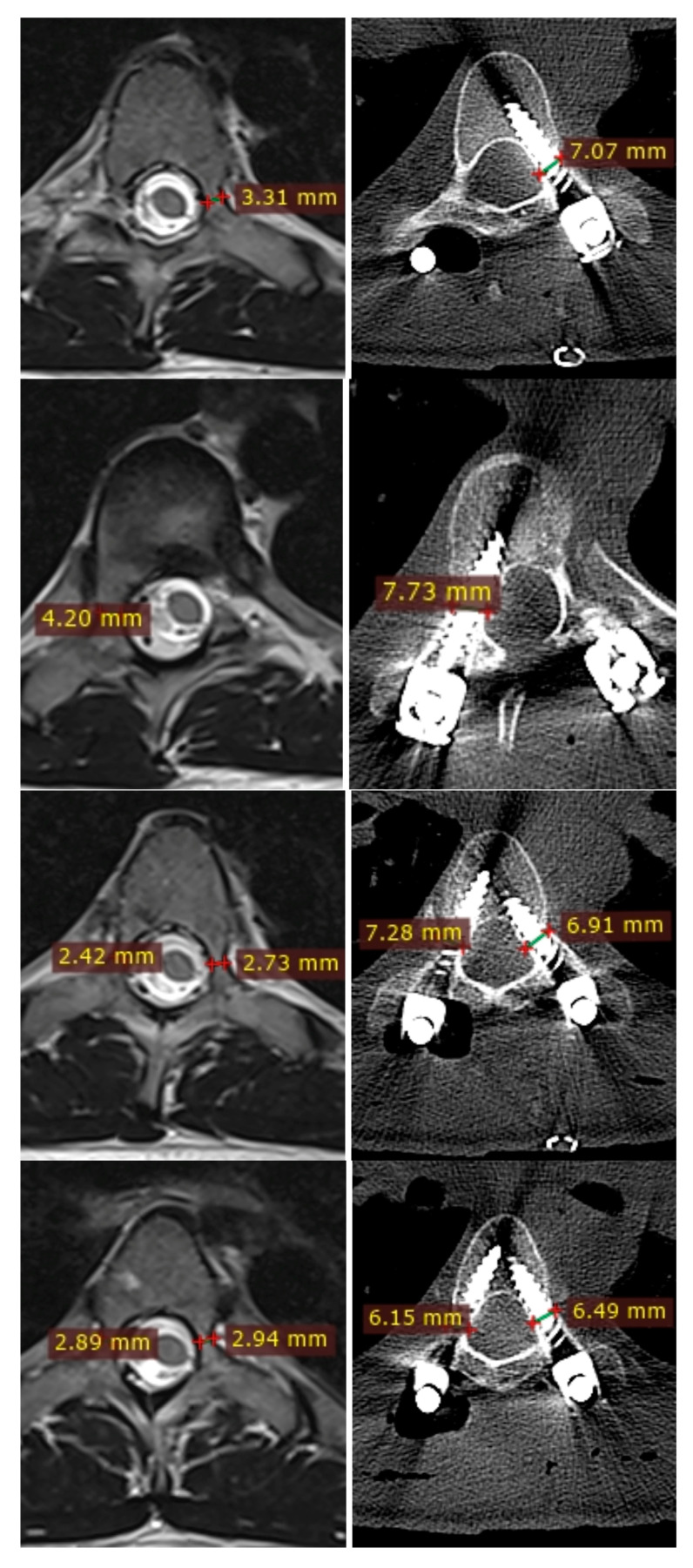

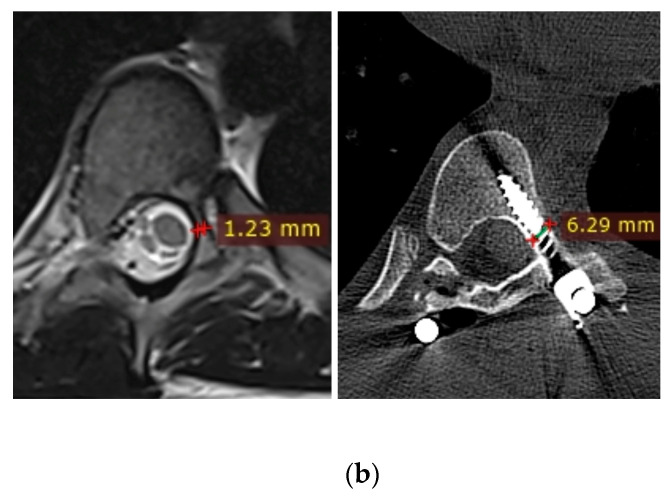
(**a**). Measurement of vertebral pedicle transverse diameter (A,B) in a magnetic resonance axial cut image. (**b**). Increasing screw size in small pedicles, showing the widening of vertebral pedicle transverse diameter without bone fracture. Preoperative images were screened via MRI and compared to the same levels with screws inserted using postoperative CT screening.

We evaluated standard posteroanterior and lateral radiographs of the entire spine in the standing position in all patients before and after surgery. Preoperative bending films were evaluated to assess the flexibility of the spine before the surgery. In MRI, spinal cord pathologies, along with pedicle morphology and types, were analyzed in all patients before surgery. All the diameters of the pedicles recorded before the operation (Figure 1a) were subjected to a comparative analysis with the measurements after the pedicle was widened after the screws had been inserted using postoperative computed tomography (Figure 1b). We measured the size of the pedicles according to the technique described in [34]. All idiopathic curves were classified according to the Lenke Classification System [35]. Postoperative CT scans were analyzed for pedicle enlargement, pedicle screw misalignment, pedicle perforation, or anterior, superior, inferior, medial, or lateral displacement, using a method described in the literature [36,37]. Before surgery, we measured the Cobb angles of the proximal thoracic, major thoracic, and lumbar curvatures in all patients, and sagittal measurements of thoracic kyphosis and lumbar lordosis were performed.

### 2.4. Statistical Analysis

We used statistical analysis software (version 10.0; StatSoft Inc., Tulsa, OK, USA) to statistically process the obtained results. ANOVA and Tukey–Kramer methods were used for the calculations; standard deviation (SD) and mean, 95% confidence interval (CI), or medians with lower and upper quartiles, or frequency, were calculated. The assumption of a normal distribution was checked visually, including using the Shapiro–Wilk test. The Mann–Whitney U test and the Kruskal–Wallis analysis in the variance rank test were used to analyze comparisons between the groups. Pearson’s correlation coefficients were calculated to assess the relationship between two numerical variables. Changes between the two time points were assessed using the McNemar test; a *p* value of <0.05 was considered statistically significant.

## 3. Results

In 230 evaluated patients, a total of 5080 pedicle screws were inserted at the T1–L5 spine levels. In the study groups, there were 28 males and 202 females, and 7954 vertebral pedicles were scanned by preoperative MRI. Detailed data from the evaluated patients before and after surgery are presented in Table 1. The morphology of pedicles analyzed before surgery via MRI screening was classified by the Watanabe scale [9] and was as follows: 55.2% type A, 33.8% type B, 4.7% type C, and 6.3% type D. The pedicle screws inserted during surgery were screened on postoperative CT and presented a placement of 52.8% in type A for Group S and 54.1% for Group L; 35.6% and 37.2% in type B, respectively, for Group S and L; 3.8% and 4.2% in type C; 7.8% and 4.5%, respectively, for Group S and L (without significant statistical differences). Postoperative lateral and medial breaches were noted (as shown in Table 2). All breaches were classified as Grade 1 or 2 and did not cause any intraoperative or postoperative complications requiring revision surgery.

**Figure 2:**
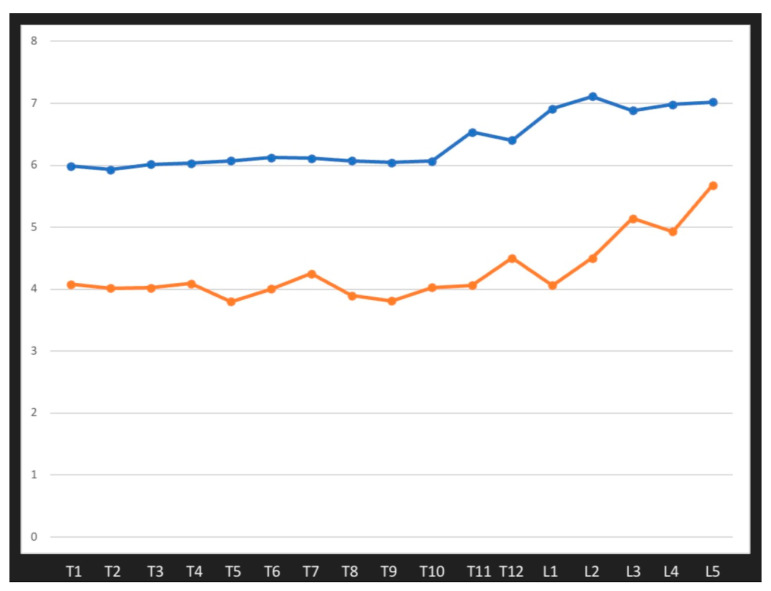
The influence of increasing size on widening vertebral pedicles for T1–L5 levels.

The mean preoperative pedicle diameter for T1–L5 vertebral pedicles ranged from 3.79 (1.44) mm to 5.68 (1.64) mm. The mean expanding diameter of pedicles after surgery for T1–L5 vertebral pedicles ranged from 1.90 (0.39) mm to 2.92 (0.28) mm, which corresponds to the extension of the pedicle diameter in the mean range of 47% (4.1)–71% (3.0), as shown in Figure 2. Detailed data for all evaluated pedicle levels are presented in Table 3.

The influence of increasing size on widening vertebral pedicles for T1–L5 levels is presented in Figure 2.

When we compared pedicle expansion between the groups, we noted a mean vertebral pedicle expansion of 49% in Group 1, 52% in Group 2, and 62% in Group 3 (N.S.). There was no statistically significant difference in breaches between Group 1 and Group 2, but there was a significant difference between Group 1 and Group 3 (*p* = 0.03) (details presented in Table 4).

When we compared the mean expansion of pedicle diameter between groups including different screw sizes, there was a mean range of increasing pedicles of between 29% (2.8) and 52% (2.8) in Group S and between 49% (3.8) and 73% (3.4) in Group L. For all levels, T1–L5, there were statistically significant differences in pedicle expansion between Group S and Group L (*p* < 0.05). There were no differences in breaches between these groups (N.S.). The comparison in both groups is presented in Table 5.

**Figure 3:**
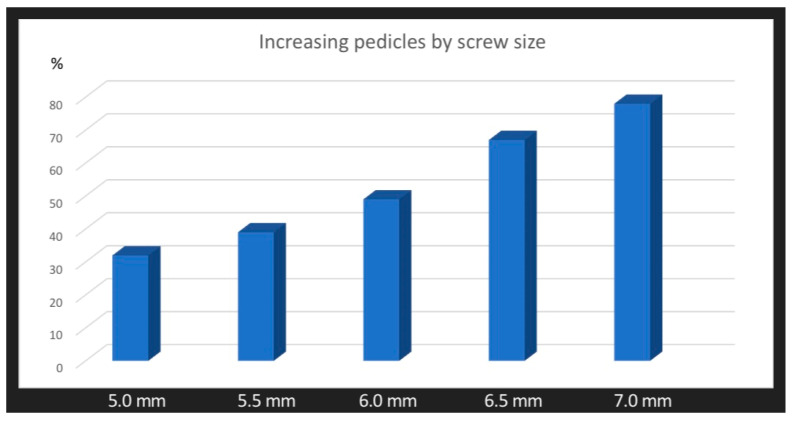
The mean widening of vertebral pedicle diameter with increasing screw diameter.

When we compared the mean expansion of the pedicle diameter between groups including different screw sizes, there was a mean range of increasing pedicle diameter of 32% (4.6) for 5.0 mm screws, 39% (5.8) for 5.5 mm screws, 49% (6.6) for 6.0 screws, 67% (8.2) for 6.5 mm screws, and 78% (8.8) for the 7.0 mm screws. For all levels, T1–L5, there were statistically significant differences in pedicle expansion between screw sizes 5.0 vs. 6.0, 5.5 vs. 6.5, 5.0 vs. 6.5, 5.0 vs. 7.0, and 5.5 vs. 7.0 (*p* < 0.05). There was no difference in breaches between these groups (N.S.). The comparison of both groups is presented in Table 6. The mean widening of the vertebral pedicle diameter using an increasing screw diameter is shown in Figure 3.

## 4. Discussion

Our retrospective study is currently the only one to have assessed the increasing diameter of pedicle screws inserted into the vertebrae and pedicle during growth. In terms of comparative assessment, this research involves the largest group of pediatric patients with spinal deformities to be studied to date. Using preoperative MRI scans, we assessed 7954 vertebral pedicles and followed the surgical treatment of 5080 pedicles after screw placement in 230 patients with spinal deformities operated on by a posterior approach and using pedicle screws. We found that using larger screws with an increased diameter resulted in pedicle dilation in the vertebrae with minimal risk of breaches. The mean expansion of vertebral pedicles in spine levels T1–L5 was in the range of 29–73% (Figure 2), without any complications. Additionally, we noted that expansion of the pedicle depends on screw diameter. Each time the size of the screw increases, the pedicle expands more; for example, for screws of 5.0 mm, the pedicle expansion had a mean value of 39%, but for screws of 6.0 mm, it had a mean value of 49%, and for 6.5 mm screws, this was 67% (Figure 3). We did not note any major complications that required revision surgery [38]. Our research shows that the use of larger-diameter pedicle screws, even in vertebral pedicles of the order of 1.5 mm, can safely expand the pedicle without breaking the medial or lateral vertebral wall (Figure 1b), and this provides better mechanical and stabilizing properties for the correction of spinal deformities, as evidenced by other authors [26,27]. According to scientific research and classification of the displacement of the pedicle screw at grades 0, 1, 2, and extra-pedicle screw insertion, for vertebral pedicles of types C and D, the obtained medial and lateral fractures constitute a safe zone for screw placement while obtaining the best biomechanical capabilities [33,34]. The larger the screw, the better the stability and fixation [19,22,39]. Unfortunately, we are not able to clearly state the limit of the gradual expansion of the pedicle using a larger screw size.

In analyzing scientific reports on the accuracy of placing pedicle screws in the vertebrae during spinal deformity surgery, many authors consider screw perforations of less than 2 mm to be acceptable and not to cause complications; this has been found to be an acceptable and safe screw position [40,41], whereas perforation tolerances (2–4 mm) are less common and recognized, but this depends on whether there are any signs of perforation of the order of 2–4 mm. Kim et al. [42] suggested and defined a “safe zone” for a safe and acceptable perforation to be less than 4 mm in diameter. Within this categorization, perforation does not interfere with arteries, veins, nerves, or organs and does not cause any symptoms. This limitation is primarily due to the ethical prohibition on testing patients’ resistance when undergoing pedicle expansion while using larger pedicle screws. Such studies have been conducted on human cadavers in the past. Cho et al. evaluated the thoracic spinal canal diameter and the effect of increasing screw diameter on vertebral pedicles in adult human cadavers aged 61–82 years old. In their analysis, the mean diameter of the largest screw before a bone fracture was visualized was 6.9 mm. Anatomically and morphologically, T12 pedicles received the largest average screws (7.9 mm), and T4 pedicles received the smallest (5.8 mm) [27]. In the current study in children and adolescents, the average diameter of the largest screw was 7.0 mm, which expanded the pedicle diameter to a mean of 78%. T2 was the pedicle with the smallest increase in diameter after screw placement, with a mean of 5.93 (0.46) mm. L2 was the pedicle with the largest increase in diameter after screw placement, with a mean of 7.11 (0.33) mm. In the study by Cho et al. [27], of the 938 pedicle screws placed, 134 bony breaches were identified in total (14.28% of pedicles), of which 133 were lateral (14.17% of pedicles) and one medial. After the insertion of 9.5 mm screws, 28 pedicles did not experience any breaches [27]. In our study, we noted 4.88% lateral breaches and 3.34% medial breaches (across all screw placements). This comparison showed the better flexibility of children’s vertebral pedicles and their potentially lower risk of complications caused by screw breaches, which has been proven in our previous study on pedicle screw accuracy [13]. There are scientific reports confirming that the medial wall of the vertebrae is the strongest, usually up to two to three times thicker than the lateral wall, and when the pedicle expands, the expansion is towards the weakest part, i.e., the lateral wall gives way first [25,27]. In other cadaver studies, the authors reported that pedicles expanding with larger screw insertions can safely be widened up to 200% and contribute 80% of cephalad–caudad stiffness and 60% of pullout strength at the screw–bone interface [16,26]. Although this can be confirmed in cadaver studies, it is difficult to obtain similar results in a group of children aged 2 to 18 years. Certainly, our study confirmed the possibility of inserting much larger screws than the width of the pedicle diameter, even in the youngest children, with a minimal risk of lateral or medial breaches and a safe extension of the pedicle of up to 78% on average. Recent retrospective studies [8] suggest that using larger screw sizes to correct the deformity can achieve significantly better correction of the three-plane deformity with a lower risk of instrumentation destabilization and loss of correction during postoperative follow-up. Analyzing the potential risk of inserting screws that are larger than the pedicles, it is possible that, if the screw is placed correctly with an accurate trajectory, the diameter of the screw may be too large for the pedicle, causing it to breach and even damage the spinal cord. However, our study showed that the majority (59%) of breaches were localized laterally. Again, these results were consistent with the fact that the center wall is thicker and stronger than the side wall. Medial breaches are mostly observed in specific types of pedicles or severe and neglected spinal deformities [13]. However, care should be taken when using a screw that is too large. We believe that insertion of a screw sized to a mean of 70% larger than the pedicle diameter is a safe method of selecting screw size, combined with assessing and analyzing other parameters of the patient, such as the etiology of deformities and past fractures, which can help in determining the weakness of the skeletal system and evaluating osteoporosis. Other researchers have previously demonstrated that an increased acceptable diameter of the pedicle screws and the accompanying increased biomechanical strength result in greater deformity correction, spinal balance restoration, and three-plane correction. Fixation strength depends primarily on the material and mechanical properties of the pedicle screw, with a large diameter significantly increasing the strength [21,28]. Surgical treatment of complex, severe, and neglected spinal deformities in children and adolescents requires complex decisions, all of which are aimed at improving the corrective capacity of the deformities, the strength of the implants used, and the acceptable postoperative stiffness of the instruments in terms of stabilized levels of the spine. In our study, we noted that the use of larger screws does not cause an increased number of statistically significant complications. The radiographic benefits reported in this large cohort study suggest that large screw sizes are safe and effective in correcting spinal deformities in children. All breaches in our study were similar to those described in the literature [11,12].

### 4.1. MRI vs. CT for Evaluating Pedicles and Pedicle Screws

It is not possible to subject a significant group of patients to a CT scan of the entire spine before and after surgery, as this carries the risk of excessive exposure to radiation. The case is slightly different with the use of a preoperative MRI for screening. There are many more possibilities to perform it, and there is no X-ray exposure during the examination [15,43]. Magnetic resonance imaging perfectly illustrates the pathologies of the spinal cord and surrounding tissues. We can also use MRI to analyze the morphology of the vertebrae and pedicles to plan implant placement. This can significantly facilitate preoperative preparation and the implementation of an appropriate operational plan. Thanks to the MRI examination, we can accurately assess the size of the pedicles and the structure of individual vertebrae, which may facilitate the insertion of pedicle screws and reduce the risk of complications. Unfortunately, there is no unequivocal opinion in the literature on the evaluation of spinal bony structures using MRI [43,44,45,46,47]. CT is regarded as being more sensitive but, on the other hand, there are many studies comparing the assessment of the bony structures of the spine with MRI and CT. Imaging of bone structures using magnetic resonance imaging (MRI) has disadvantages, consisting of a less clear scan of cortical bony structures [43]. In a study by Duchaussoy et al., all measurements made with MRI underestimated the minimum width of the pedicle transverse diameter by about 10% compared with computed tomography [15]. This difference is not significant enough to substantially increase the risk of pedicle fracture, provided the screw placement is otherwise correct [15]. The authors concluded that MRI is a valuable alternative to CT for preoperative pedicle measurements in patients with spinal deformities requiring surgical treatment. CT scans should no longer be used for this purpose due to the radiation dose a child or teenager receives. Screening of pedicle morphology and width can be performed by MRI without compromising the safety of the procedure [15]. Considering the results of our study, comparing the preoperative assessment of vertebrae with MRI and postoperative CT, and referring to previous research [15], an MRI measurement error of about 10% is not a significant impediment. With a 5 mm pedicle, 10% is underestimating its actual size by 0.5 mm. We know from human cadaver studies [26,27] and from the current study that the pedicle is flexible and expandable, with a range up to 78% (from this study) and up to 200% (from cadaveric studies). Some scientific reports note [45,48] that incorrect placement of the pedicle screw occurs in cases of abnormal anatomy and morphology of the pedicles, and, in this case, MRI has poor diagnostic value in identifying dysplastic pedicles of types C and D. On the other hand, in terms of morphology and epidemiology, the prevalence of these types of pedicles is low; these pedicles are often located in the apical, concave (usually left) side or in the upper part of the thoracic spine and in severe scoliosis [45]. In our opinion, this finding requires more attention when inserting screws and possibly the use of alternative methods, such as inserting screws in-out-in through the pedicle or the use of hooks or laminar tapes/bands. To minimize the risk of error when evaluating the epiphyses with preoperative MRI, care should be taken to ensure that MRI scans are of high quality, with an axial slice thickness of 4 mm and without gaps [45].

In another study comparing screening CT and MRI, the authors concluded that pedicle screw diameter measurements were more accurate using CT images compared with MRI images, but when using MRI images, the surgeon should be aware of the differences in screw length (about 2 mm) and pedicle diameter (about 0.5 mm) compared with CT in order to avoid intra- and postoperative risks [46]. In contrast, other authors [47] prove that preoperative MRI is suitable for measuring pedicle size with sufficient reliability during preoperative planning to predict vertebral pedicle dimensions, as revealed by preoperative CT imaging. Based on the analysis of this study, preoperative MRI and CT images provide equivalent performance in measuring vertebral anatomy, although the predicted diameter of the pedicle on MRI may be less than that on CT by approximately <0.5 mm, confirming other reports cited above. Accordingly, preoperative MRI can be considered sufficient, non-irradiating, and able to produce the data necessary to perform pedicle and vertebral size measurements.

### 4.2. Limitations

Our work is a retrospective study, but we evaluated a large group of patients and implanted pedicle screws. During the analysis of patients, data were limited to available medical records. The strength of our study was the large number of analyzed pedicles, which we were able to investigate in detail in the preoperative MRI examination, and we could then compare how the same pedicles looked after screw insertion using the postoperative CT scan. All patients were operated on by experienced spinal surgeons with many years of work experience, including the insertion of transpedicular screws. All patients were prepared for surgery by performing a preoperative MRI of the whole spine. After surgery, we performed CT screening for patients, and we selected patients who had both preoperative MRI and postoperative CT scans for comparison of vertebral pedicle morphology and anatomic measurements. In our opinion, further research is indicated to more accurately assess and understand the mechanism of pedicle size expansion and its effect on biomechanical properties and the correction of spinal deformities.

## 5. Conclusions

Our study confirms that a wide range of expansion of the vertebral pedicle is possible during screw insertion, including up to 78%, with a low risk of lateral or medial breaches and without an increased risk of intraoperative and postoperative complications. The larger the diameter of the screw that is inserted into the pedicle, the more the pedicle expands. Pedicle measurements by preoperative MRI may be helpful in providing sufficient reliability for preoperative planning.

## Figures and Tables

**Table 1:** Patient demographics.

Parameter	Group 1 (n = 54)	Group 2 (n = 61)	Group 3 (n = 115)
Age (years, range) Mean (SD) age at surgery	2–5 4.2 (0.8)	6–10 7.6 (2.2)	11–18 13.8 (3.2)
*p* value	*p* = 0.33 (1 vs. 2)	*p* = 0.41 (2 vs. 3)	*p* = 0.27 (1 vs. 3)
Sex			
Male	8	11	9
Female	46	50	106
Etiology:			
Congenital (n)	8	6	11
Neuromuscular (n)	6	8	9
Syndromic (n)	5	6	7
Idiopathic (n)	35	41	88
Main curve magnitude (degrees, range) preoperative Mean (SD) values	70–125 88 (13.2)	65–128 85 (18.9)	50–165 81 (29.2)
*p* value	*p* = 0.13 (1 vs. 2)	*p* = 0.09 (2 vs. 3)	*p* = 0.08 (1 vs. 3)
Main curve magnitude (degrees, range) postoperative Mean (SD) values	21–81 35 (18)	25–67 32 (15)	5–88 28 (22)
*p* value	*p* = 0.83 (1 vs. 2)	*p* = 0.66 (2 vs. 3)	*p* = 0.78 (1 vs. 3)
Thoracic kyphosis (degrees, range) preoperative Mean (SD) values	21–105 49 (26)	31–121 52 (28)	17–154 78 (48)
*p* value	*p* = 0.69 (1 vs. 2)	*p* = 0.52 (2 vs. 3)	*p* = 0.79 (1 vs. 3)
Thoracic kyphosis (degrees, range) postoperative Mean (SD) values	18–72 47 (14)	26–68 49 (12)	15–82 36 (17)
*p* value	*p* = 0.26 (1 vs. 2)	*p* = 0.29 (2 vs. 3)	*p* = 0.18 (1 vs. 3)
Number of screw placements	689	1251	3140
Blood loss (range, cc) Mean (SD) values	120–620 282 (148)	90–820 547 (328)	280–1800 680 (392)
*p* value	*p* = 0.88 (1 vs. 2)	*p* = 0.91 (2 vs. 3)	*p* = 0.48 (1 vs. 3)
Operation time (range, min.) Mean (SD) values	75–420 212 (68)	88–880 244 (72)	152–820 348 (168)
*p* value	*p* = 0.39 (1 vs. 2)	*p* = 0.28 (2 vs. 3)	*p* = 0.53 (1 vs. 3)

**Table 2:** The percentage representation of the type of pedicle screws.

Pedicle Type	All Pedicles Preop	Group S Postop	Group L Postop	Postop Lateral Breaches (n = 248)	Postop Medial Breaches (n = 170)
A	55.2%	52.8%	54.1%	22 (0.43%)	15 (0.29%)
N.A.		*p* = 0.471		*p* = 0.547	
B	33.8%	35.6%	37.2%	39 (0.76%)	22 (0.43%)
N.A.		*p* = 0.377		*p* = 0.113	
C	4.7%	3.8%	4.2%	89 (1.75%)	55 (1.08%)
N.A.		*p* = 0.213		*p* = 0.08	
D	6.3%	7.8%	4.5%	98 (1.92%)	78 (1.53%)
N.A.		*p* = 0.188		*p* = 0.09	

Statistical comparisons were performed using the Kruskal–Wallis test, two-sided *t*-test, or Wilcoxon test; *p* < 0.05 for all.

**Table 3:** The mean diameter of pedicles before surgery, after screw insertion, and the mean expanding pedicle diameter after screw insertion.

Levels, Patients n = 230	Mean Pedicle Preop N = 7954	Mean Pedicle Postop N = 5080	Mean Expanding (mm)	Mean Expanding %
T1	4.07 (0.96)	5.98 (0.38)	1.90 (0.32)	48% (3.4)
T2	4.01 (1.2)	5.93 (0.46)	1.92 (0.39)	47% (4.1)
T3	4.02 (1.92)	6.01 (0.27)	2.01 (0.26)	50% (2.7)
T4	4.09 (1.76)	6.02 (0.26)	2.04 (0.28)	49.5% (2.8)
T5	3.79 (1.44)	6.07 (0.23)	2.55 (0.22)	68% (2.2)
T6	4.00 (1.83)	6.12 (0.43)	2.44 (0.18)	61% (2.1)
T7	4.25 (1.55)	6.12 (0.44)	2.18 (0.21)	52% (2.2)
T8	3.90 (1.58)	6.07 (0.36)	2.11 (0.29)	54% (3.2)
T9	3.81 (1.73)	6.04 (0.36)	2.51 (0.33)	66% (3.8)
T10	4.02 (1.59)	6.06 (0.38)	2.21 (0.32)	55% (3.6)
T11	4.06 (1.67)	6.53 (0.37)	2.53 (0.39)	62% (3.7)
T12	4.18 (1.48)	6.40 (0.45)	2.39 (0.36)	57% (3.7)
L1	4.06 (1.59)	6.91 (0.22)	2.88 (0.29)	71% (3.0)
L2	4.5 (1.38)	7.11 (0.33)	2.67 (0.28)	59% (2.9)
L3	5.14 (1.2)	6.88 (0.38)	2.89 (0.34)	56% (3.3)
L4	4.93 (1.5)	6.98 (0.24)	2.92 (0.28)	59% (2.7)
L5	5.68 (1.64)	7.02 (0.34)	2.68 (0.29)	47% (3.0)

**Table 4:** The mean diameter of pedicles before surgery, after screw insertion, and the mean expanding pedicle diameter after screw insertion.

Variable	Group 1 (n = 54)	Group 2 (n = 61)	Group 1 vs. Group 2, *p*	Group 3 (n = 115)	Group 2 vs. Group 3, *p*	Group 1 vs. Group 3, *p*
Mean pedicle expansion%	49% (2.5)	52% (3.5)	*p* = 0.43	62% (3.8)	*p* = 0.12	*p* = 0.07
Breaches lat. by age	61 (1.2%)	68 (1.33%)	*p* = 0.82	119 (2.34%)	*p* = 0.92	*p* = 0.03
Breaches med. by age	52 (1.02%)	47 (0.92%)	*p* = 0.79	71 (1.4%)	*p* = 0.79	*p* = 0.12

Statistical comparisons were performed using the Kruskal–Wallis test, two-sided *t*-test, or Wilcoxon test; *p* < 0.05 for all.

**Table 5:** The mean diameter of pedicle expansion after surgery and breaches during the groups.

Level (n = 5080 Inserted Screws)	Mean Pedicle Expansion Postop (%) Group S	Postop Breaches (Medial/Lateral) Group S	Mean Pedicle Expansion Postop (%) Group L	Postop Breaches (Medial/Lateral) Group L	Expansion L. vs. S, *p*	Breaches L vs. S, *p*
T1	34% (2.8)	3/8	50% (2.8)	3/8	*p* = 0.031	N. S.
T2	36% (3.2)	2/7	49% (3.4)	3/7	*p* = 0.038	N. S.
T3	40% (2.8)	3/9	52% (3.2)	4/9	*p* = 0.042	N. S.
T4	36% (3.1)	4/9	52.5% (3.2)	4/10	*p* = 0.022	N. S.
T5	50% (4.1)	5/9	70% (2.2)	5/10	*p* = 0.001	N. S.
T6	42% (3.2)	4/9	61% (2.6)	6/9	*p* = 0.041	N. S.
T7	35% (2.8)	6/10	54% (2.6)	8/12	*p* = 0.039	N. S.
T8	50% (3.6)	6/11	55% (3.5)	9/12	*p* = 0.039	N. S.
T9	52% (2.8)	5/9	67% (3.4)	9/10	*p* = 0.012	N. S.
T10	46% (3.2)	7/8	57% (3.8)	8/9	*p* = 0.019	N. S.
T11	45% (1.9)	9/9	65% (3.9)	9/8	*p* = 0.011	N. S.
T12	39% (4.2)	4/5	59% (3.2)	6/4	*p* = 0.039	N. S.
L1	38% (3.8)	4/6	73% (3.4)	7/7	*p* = 0.001	N. S.
L2	34% (2.6)	3/8	63% (2.8)	6/7	*p* = 0.001	N. S.
L3	37% (3.2)	3/7	59% (3.8)	8/8	*p* = 0.012	N. S.
L4	31% (3.2)	2/1	62% (3.8)	4/1	*p* = 0.001	N. S.
L5	29% (2.8)	1/1	49% (3.8)	1/1	*p* = 0.031	N. S.

Statistical comparisons were performed using the Kruskal–Wallis test, two-sided *t*-test, or Wilcoxon test; *p* < 0.05 for all.

**Table 6:** The mean diameter of pedicle expansion after surgery and breaches in different groups of screw sizes.

Variable	5.0 mm	5.5 mm	6.0 mm	6.5 mm	7.0 mm
Mean pedicle expansion % by screw size	32% (4.6)	39% (5.8)	49% (6.6)	67% (8.2)	78% (8.8)
5.0 vs. 6.0 *p* = 0.03	5.0 vs. 5.5 *p* = 0.661	5.5 vs. 6.0 *p* = 0.227	6.0 vs. 6.5 *p* = 0.113	6.5 vs. 7.0 *p* = 0.121	5.5 vs. 6.5 *p* = 0.03
5.0 vs. 6.5 *p* = 0.01		5.0 vs. 7.0 *p* = 0.01		5.5 vs. 7.0 *p* = 0.01	6.0 vs. 7.0 *p* = 0.01
Breaches lat. by screw size	49 (0.96%)	49 (0.96%)	48 (0.94%)	55 (1.08%)	47 (0.92%)
Breaches med. by screw size	28 (0.55%)	32 (0.63%)	34 (0.67%)	39 (0.76%)	37 (0.72%)
Total breaches	77 (1.51%)	81 (1.59%)	82 (1.61%)	94 (1.85%)	84 (1.65%)
*p* Breaches lat. vs. med.	*p* = 0.39	*p* = 0.221	*p* = 0.132	*p* = 0.07	*p* = 0.119

Statistical comparisons were performed using the Kruskal–Wallis test, two-sided *t*-test, or Wilcoxon test; *p* < 0.05 for all.

## Data Availability

Not applicable.
